# Supplementary figures and images for: Molecular evidence that the Channel Islands populations of the orange-crowned warbler (Oreothlypis celata; Aves: Passeriformes: Parulidae) represent a distinct evolutionary lineage
Source: PeerJ. 2019 Aug 6;7:e7388. doi: 10.7717/peerj.7388 (PMC6688592; doi:10.7717/peerj.7388)

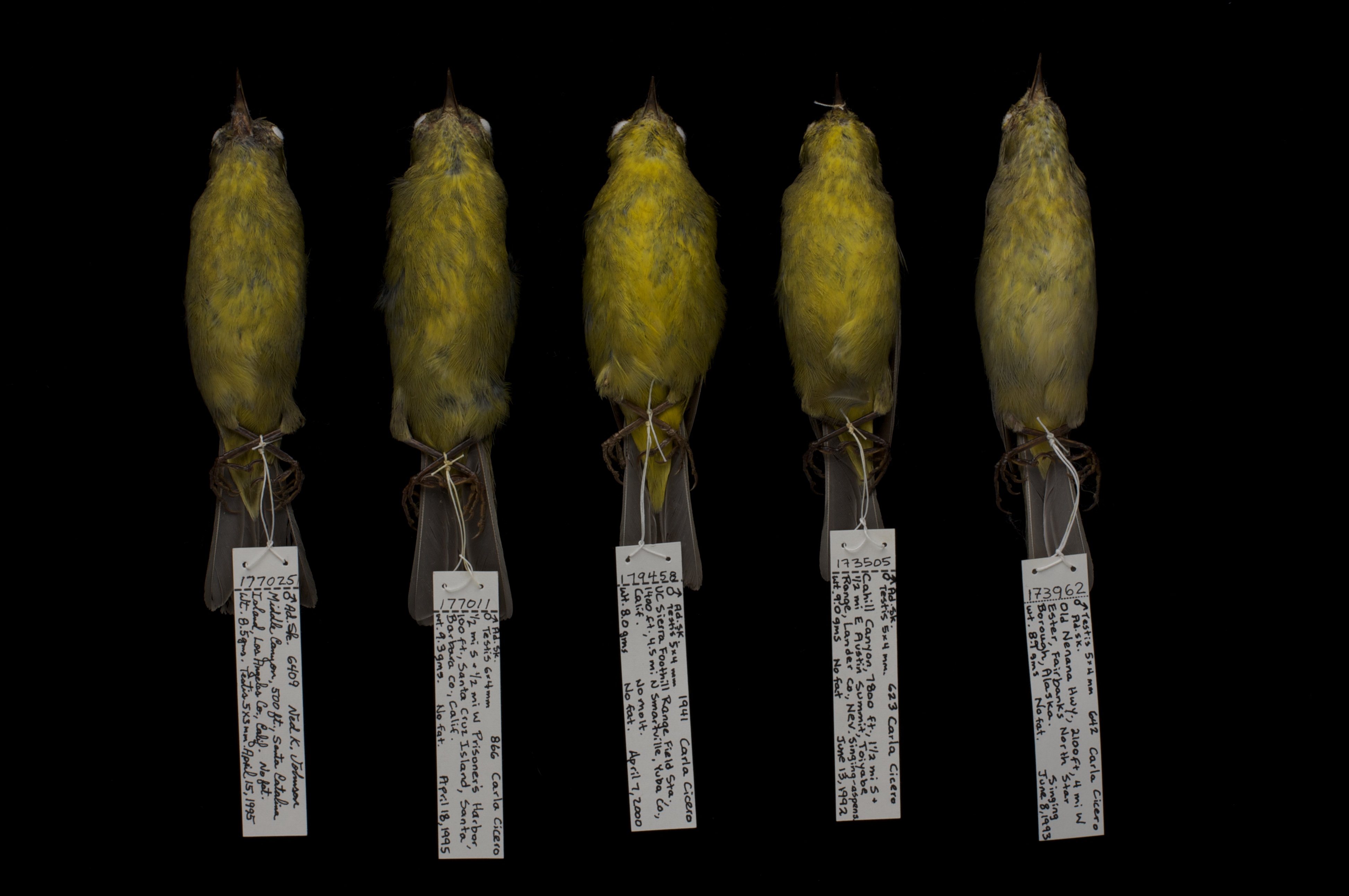

Supplement: Figure S1 — This is a ventral view of five Oreothlypis celata museum skins. From left to right, the specimens are MVZ:Bird:177025, MVZ:Bird:177011, MVZ:Bird:179458, MVZ:Bird:173505, and MVZ:Bird:173962. These represent the four recognized subspecies with representatives of O. c. sordida from both the southern and northern Channel Islands. From left to right, the specimens are an O. c. sordida from Santa Catalina Island; an O. c. sordida from Santa Cruz Island; an O. c. lutescens from interior northern California; an O. c. orestera from Nevada; and an O. c. celata from Fairbanks, Alaska. Image taken by Anand Varma, reproduced with permission. [file peerj-07-7388-s005.jpg]

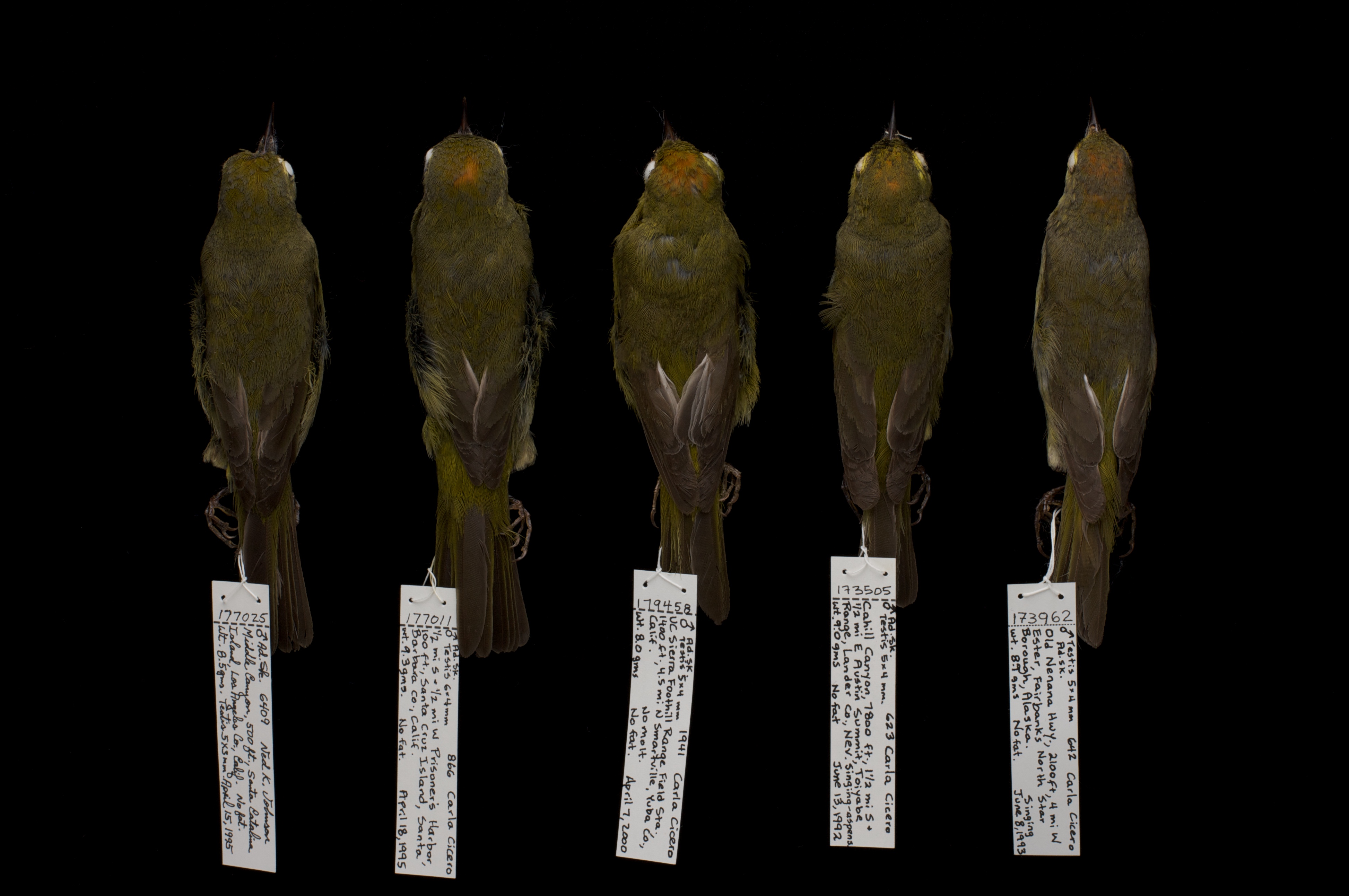

Supplement: Figure S2 — This is a dorsal-view of the same five specimens seen in Fig. S1. Image taken by Anand Varma, reproduced with permission. [file peerj-07-7388-s006.jpg]

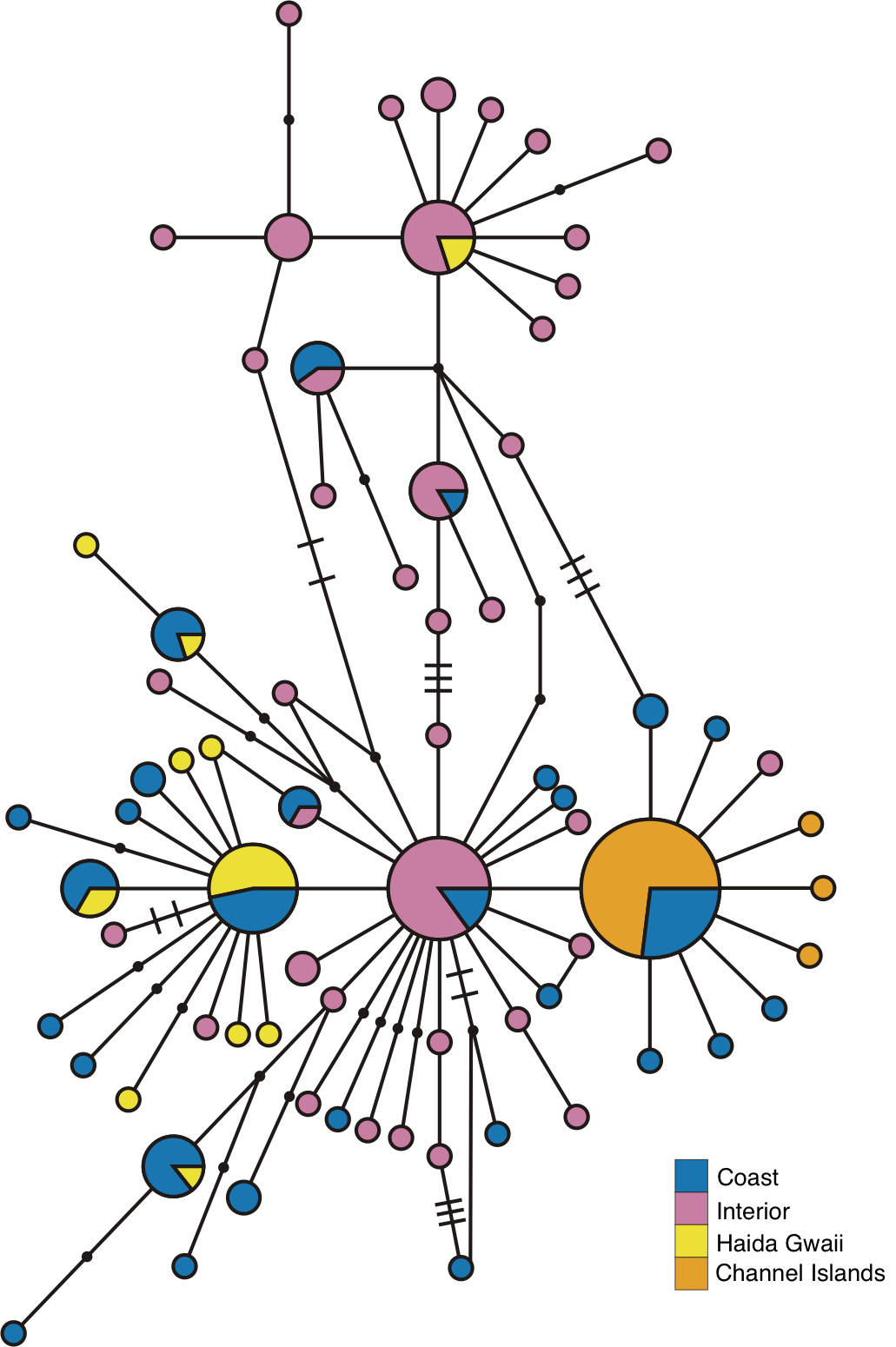

Supplement: Figure S3 — This is the ND2 haplotype network colored by samples’ grouping into coast and interior populations. See Fig. 3 for the haplotype numbers that correspond with the numbers in Table S1. The size of each circle is proportional to the number of individuals with that haplotype. Lines connect haplotypes that differ by one mutation. Dots represent inferred haplotypes. Hash marks indicate the number of mutations between haplotypes separated by more than one mutation. [file peerj-07-7388-s007.png]

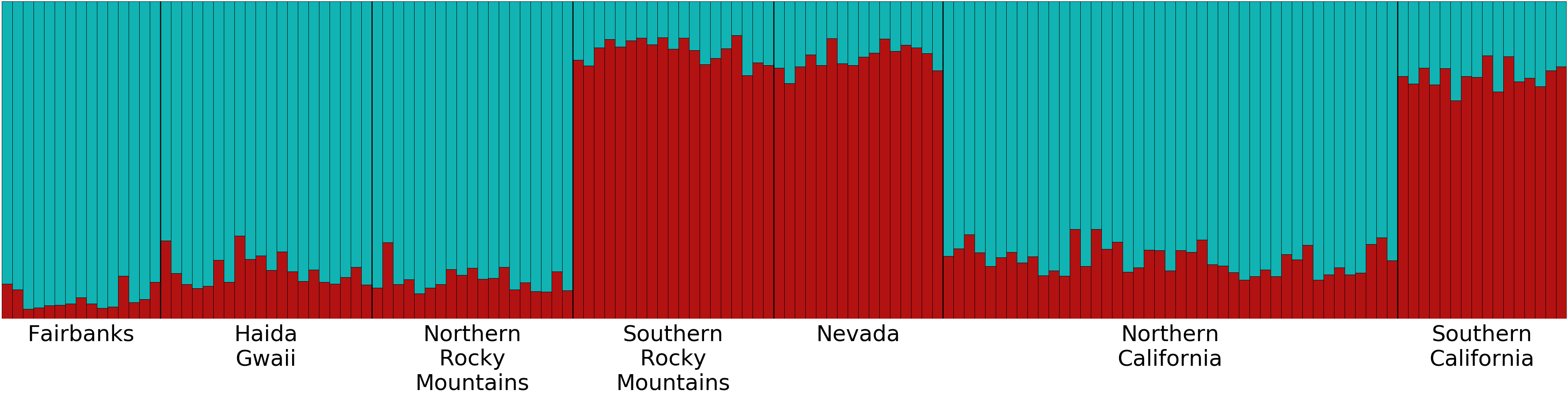

Supplement: Figure S4 — This figure depicts the ancestry of each individual in the two genetic clusters identified by Structure within the seven labeled populations after we excluded the Channel Islands population. Different colors represent the two genetic clusters identified by Structure. Each vertical bar represents an individual Oreothlypis celata. The height of each color in a given bar illustrates the proportion of ancestry derived from each genetic cluster for that individual. [file peerj-07-7388-s008.png]
